# The Prevalence of Human Papilloma Virus in Turkish Women

**DOI:** 10.3390/jcm15166323

**Published:** 2026-08-16

**Authors:** Göktürk Han Doğan, Ceren Sağlam Purut, İbrahim Gülhan, İlayda Gercik Arzık, Abdurrahman Hamdi İnan, Muzaffer Sancı, Rumeysa Nur Çağıran, Yunus Emre Purut

**Affiliations:** 1 Department of Gynecologic Oncology, İzmir Tepecik Training and Research Hospital, 35100 İzmir, Türkiye; purutemreyunus@hotmail.com; 2 Department of Perinatology, İzmir Atatürk Training and Research Hospital, 35360 İzmir, Türkiye; drcerensaglam@yahoo.com; 3 Department of Obstetrics and Gynecology, İzmir Tepecik Training and Research Hospital, 35100 İzmir, Türkiye; drigulhan@yahoo.com (İ.G.); ahamdiinan@gmail.com (A.H.İ.); rumeysacagiran98@gmail.com (R.N.Ç.); 4 Department of Perinatology, İzmir City Hospital, 35540 İzmir, Türkiye; ilgercik@gmail.com; 5 Department of Gynecologic Oncology, İzmir City Hospital, 35540 İzmir, Türkiye; drsanci@yahoo.com

**Keywords:** HPV prevalence, human papilloma virus, multiple HPV infection

## Abstract

**Background/Objectives**: The prevalence of Human Papilloma Virus (HPV) in Türkiye may vary from that reported in other countries. Determining the age-specific distribution of HPV infection and its overall prevalence in all tested individuals, supported by a large sample size, would provide valuable insights for HPV prevention programs. This study aims to determine the regional prevalence of HPV infection. **Methods**: This is a retrospective study containing data from 52,930 patients who underwent HPV testing at a tertiary hospital between 2016 and 2024. Patients aged 21 to 65 were included in the study. Target DNA amplification by polymerase chain reaction (PCR) and nucleic acid hybridization techniques were performed for the identification of HPV. Overall prevalence, age-specific prevalence, and genotype distribution were examined. **Results**: Analysis of genotype distribution revealed that isolated HPV16 positivity was 4.0% (*n* = 2118), isolated HPV18 positivity was 1.0% (*n* = 528), and isolated other high-risk (HR) HPV types (excluding HPV16 and HPV18) were detected in 10.8% (*n* = 5693) of patients. The co-positivity rates were as follows: HPV16 and HPV18 in 0.4% (*n* = 237), HPV16 and HR-HPV other in 2.3% (*n* = 1197), HPV18 and HR-HPV other in 1.0% (*n* = 513), and HPV16, HPV18, and HR-HPV other in 0.3% (*n* = 145). **Conclusions**: This study highlights that HPV positivity is higher among individuals aged 21–24 years in our population, the overall prevalence is broadly consistent with the range reported in previous Turkish and international studies, and the pooled other HR-HPV group was more frequent than HPV16 or HPV18 individually.

## 1. Introduction

Human papillomavirus (HPV) is a common infectious agent worldwide and the primary cause of cervical cancer, particularly through its high-risk types. In addition to cervical cancer, HPV is also associated with cancers of the vulva, anus, oropharynx, and penis [1]. HPV is classified into two groups: high-risk and low-risk types [2]. The International Agency for Research on Cancer (IARC) has identified HPV types 16, 18, 31, 33, 35, 39, 45, 51, 52, 56, 58, and 59 as high-risk genotypes associated with cervical cancer [3]. Approximately 70% of cervical cancer cases result from persistent infection with HPV types 16 and 18, as reported in a review of HIV-infected and HIV-uninfected women [4]. HPV-related infections are asymptomatic in the vast majority of cases, leading to significant delays in diagnosis. These diagnostic delays may require treatments of varying complexity, resulting in substantial workforce loss and financial burden.

In our country, HPV screening was restructured in 2014, and the current screening program is based on HPV genotyping and cytological evaluation. On average, approximately 30,000 tests are performed weekly and around 1 million tests annually in Türkiye [5]. With the update of the screening method, the HPV positivity rate increased from 3% in 2012 to 35% in 2017 [5]. Similar HPV positivity rates have been reported in other countries as well, with rates reaching up to 44.5% in China [6] and approximately 32% in Eastern Africa [7]. It is predicted that with vaccination and widespread screening, 12.5–13.5 million cervical cancer cases could be prevented by 2070, and cervical cancer could be virtually eliminated by the end of the century [8]. In this study, we analyzed the results of regional screening programs, HPV positivity rates, and their distribution by age. Based on these data, our study aims to provide region-specific evidence to guide the optimal timing for initiating screening, evaluate efficacy and cost-effectiveness through intergroup comparisons prior to vaccination, and inform prophylactic vaccination strategies according to the distribution of region-specific HPV genotypes.

## 2. Materials and Methods

Between December 2016 and December 2024, a total of 52,930 female patients aged 21–65 were included in this study. All participants were examined either at the gynecologic oncology or the obstetrics and gynecology outpatient clinics of Tepecik Training and Research Hospital. In accordance with ASCCP guidelines, the 21–65 age group was included in the study. In addition to routine cervical cancer screening, HPV testing was also performed for patients presenting with persistent infections despite treatment, postcoital bleeding, or genital lesions. Patients younger than 21 years or older than 65 years were excluded from the study. In our study, only patients’ first admissions were included. Patients who were already under follow-up due to existing HPV positivity and who underwent control sampling were excluded from the study. As the study was defined as retrospective, HPV genotyping was performed at the time of sampling. No additional analyses were conducted retrospectively on stored materials from the selected cohort.

HPV tests were analyzed using the cobas 4800 HPV Test (Roche Molecular Systems, Inc., Pleasanton, CA, USA). The Cobas 4800 is an in vitro qualitative assay for the detection of human papillomavirus. This test uses polymerase chain reaction (PCR) and nucleic acid hybridization for target DNA amplification to identify high-risk HPV types. This test specifically identifies HPV types 16 and 18 and is designed to identify other clinically significant high-risk types (31, 33, 35, 39, 45, 51, 52, 56, 58, 59, 66, and 68). These additional genotypes are reported by the assay as a single pooled “other high-risk” result rather than individually; among them, HPV66 and HPV68 are classified by the IARC as Group 2B and Group 2A carcinogens, respectively. The minimum sample volume required for detection is 1000 µL. It utilizes the β-globin gene as an internal control to verify specimen adequacy and to detect amplification inhibition, thereby identifying invalid results. The manufacturer’s instructions state that the test does not cross-react with low-risk HPV genotypes, thereby ensuring that positive results are clinically significant. The clinical performance of the Cobas 4800 HPV test has been established in large-scale screening validation studies.

Statistical analyses were performed using the Statistical Package for the Social Sciences (SPSS) version 25.0 (IBM Corp., Armonk, NY, USA). Categorical variables were summarized as frequencies and percentages, while continuous variables were presented as mean ± standard deviation or, where appropriate, as median and interquartile range (25–75th percentiles). The Chi-square test was used to compare categorical variables. A *p*-value of <0.05 was considered statistically significant for all analyses.

This study was approved by the Non-Interventional Research Ethics Committee of İzmir Tepecik Training and Research Hospital (decision no. 2025/07-07). The research was conducted in accordance with the principles of the Declaration of Helsinki. As this was a retrospective study, informed consent was not obtained from the patients. All screening tests were performed in the hospital’s laboratories in compliance with biosafety guidelines.

## 3. Results

Between 2016 and 2024, a total of 52,930 female patients were included in the study. Analysis of demographic data revealed that 4% (*n* = 2140) of the patients were aged 21–24 years, 7.5% (*n* = 3975) were aged 25–29 years, and 88.4% (*n* = 46,815) were aged 30–65 years. The mean age of the participants was 42.7 ± 10.5 years (median: 42) (Table 1).

Overall, high-risk HPV (HR-HPV) positivity was detected in 19.7% (*n* = 10,431; 95% CI 19.4–20.0) of the patients. The patients were categorized as follows: HPV16 positive only, HPV18 positive only, high-risk HPV other (HR-HPV other) positive (excluding HPV16 and HPV18), HPV16 and HPV18 co-positive, HPV16 and HR-HPV other co-positive, HPV18 and HR-HPV other co-positive, and HPV16, HPV18, and HR-HPV other triple-positive.

Analysis of genotype distribution showed that HPV16 positivity alone was observed in 4.0% (*n* = 2118) of cases, HPV18 positivity alone in 1.0% (*n* = 528), and other HR-HPV positivity alone in 10.8% (*n* = 5693). Co-infection rates were as follows: HPV16 and HPV18 co-infection in 0.4% (*n* = 237), HPV16 and HR-HPV other in 2.3% (*n* = 1197), HPV18 and HR-HPV other in 1.0% (*n* = 513), and HPV16, HPV18, and HR-HPV other triple co-infection in 0.3% (*n* = 145) (Table 2).

For further statistical analysis of the relationship between genotype positivity rates and age, patients were stratified into three groups: 21–24 years, 25–29 years, and ≥30 years. It was observed that patients aged 30 years and older had significantly lower positivity rates for HPV16, HPV18, HR-HPV other, HPV16 and HPV18 co-positivity, HPV16 and other HR-HPV co-positivity, and HPV16, HPV18, and HR-HPV other triple-positivity, as well as for overall positivity, compared with the younger age groups (*p* < 0.05). Consistent with this trend, the co-positivity rate of HPV18 and HR-HPV other was also significantly higher in the younger age groups (*p* < 0.001) (Table 3).

Across all age groups, HR-HPV other was found to be more common than any other HPV genotype.

**Figure 1:**
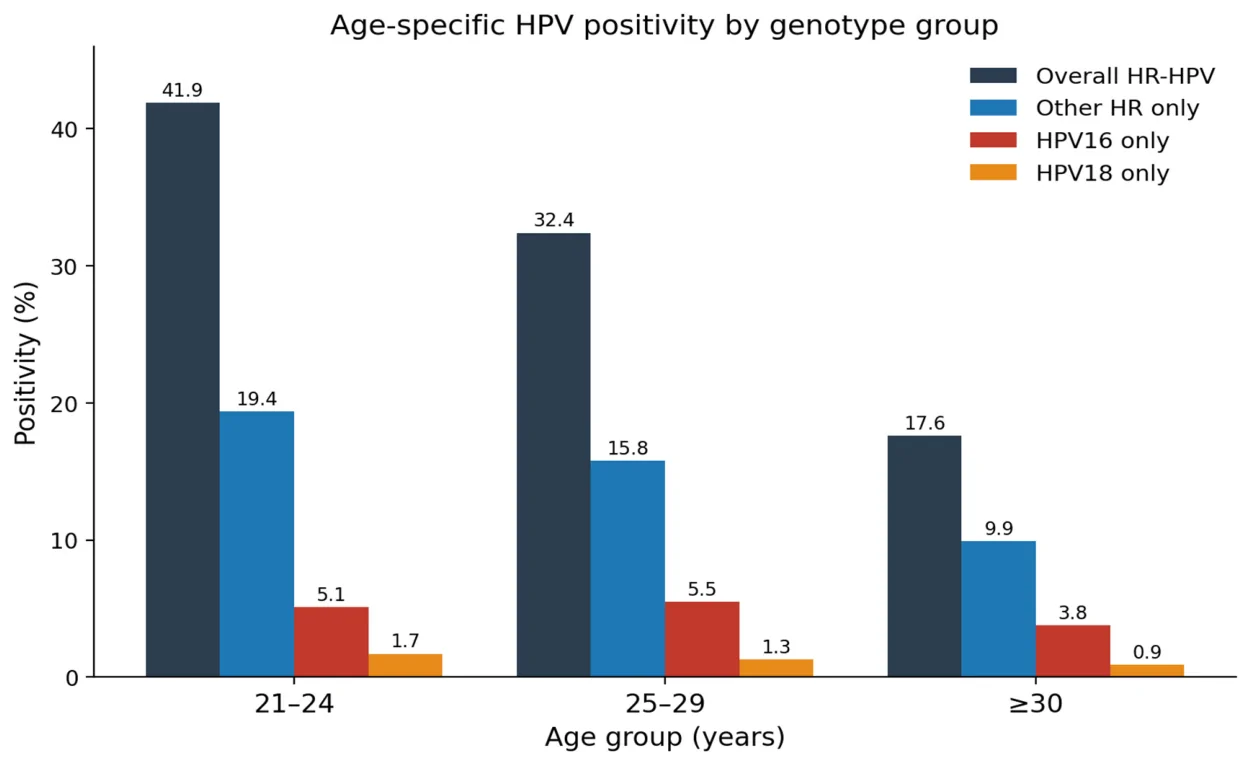
Age-specific HPV positivity by genotype group (based on the values in Table 3).

The age-specific positivity across genotype groups is illustrated in Figure 1. Point prevalence estimates with 95% confidence intervals for every genotype category and age group are provided in Table S1 (Supplementary Materials).

HPV16 and HPV18, the genotypes most strongly associated with cervical cancer, were detected in any combination in 4738 women (9.0%). The reason this figure does not match the sum of the numbers in Table 4 is that the simultaneous positivity of HPV 16 and 18 is included in both groups.

## 4. Discussion

In our study, we evaluated the positivity rates of HPV genotypes in our region and compared the distribution of positive results across different age groups. HPV positivity was detected in 19.7% (*n* = 10,431) of the patients. Other high-risk HPV (HR-HPV) types were the most common detected genotypes among all positivity rates.

To our knowledge, this represents one of the largest single-center HPV prevalence cohorts reported from Türkiye, providing age-specific genotype distribution over a multi-year period.

In a large-scale study conducted in Chongqing, China, involving 108,863 patients, the HPV positivity rate was reported as 30.14% [9], which is notably higher than the rate observed in our study. Conversely, a retrospective study by Brotherton et al. in Australia, which included 116,052 patients, reported a much lower HPV positivity rate of 9.25% [10]. A meta-analysis published in 2010, which included data from 194 studies and over 1 million patients, reported a global HPV positivity rate of 11.74%. In the same analysis, the prevalence rate for Eastern Europe—a region that includes our country—was 21.4% [11]. Furthermore, consistent with our findings, the highest positivity rates in this meta-analysis were observed in women under the age of 25.

Globally, several studies have demonstrated that HPV prevalence among women ranges from 2% to 44% [12]. These data indicate significant variability in HPV positivity rates between countries. For example, a study by Bakhshani et al. conducted in Iran with 1230 participants reported an overall HPV positivity rate of 31.8% [13]. In Hungary, two different studies reported HPV positivity rates of 5% and 21%, respectively, despite having similar mean ages among participants [14]. Such discrepancies underscore the considerable heterogeneity of data on HPV prevalence. Studies conducted in our country have also demonstrated a wide range of prevalence rates. İnal et al. reported an HPV prevalence of 2% [15], Özçelik et al. reported 6% [16], and Taşkın et al., in a study involving 5406 participants, reported an HPV positivity rate of 9.1% [17]. Another study conducted in Türkiye with 10,152 women reported a positivity rate of 10.9% [18]. Across various studies in Türkiye, HPV prevalence has been reported to range from 2.1% to 25.7% [18,19].

Several factors may explain the variation in HPV prevalence across studies. Methodological differences are prominent: assays such as the one used here report twelve additional high-risk genotypes as a single pooled result, which increases the apparent detection rate relative to assays restricted to HPV16/18. Population composition also contributes; in addition to routine screening, our cohort included women tested for clinical indications (persistent infection, postcoital bleeding, or genital lesions), which may enrich for positivity. Biological and epidemiological factors—age at sexual debut, number of partners, gradual immune clearance with age, and differences in screening-initiation age—further account for cross-country and cross-study differences. The predominance of the pooled other-high-risk group over HPV16/18 in our cohort is expected in a screening population and is consistent with previous prevalence studies, whereas HPV16 and HPV18 predominate in invasive cervical cancer [20].

The HPV positivity rate found in our study falls within the wide range reported in the literature; it is higher than in some European populations (e.g., Spain) and comparable to or lower than in others (e.g., Denmark). In a multicenter study conducted in 14 European countries, the lowest prevalence was observed in Spain (2.2%) and the highest in Denmark (22.8%) [21]. A study conducted in Italy reported an HPV positivity rate of 14% [22]. Similar to our findings, a previous study reported that HPV prevalence is highest among women under the age of 25, with rates ranging from 32% to 64% [23].

This study has additional limitations. Its retrospective design and reliance on laboratory records precluded the collection of individual risk factors such as vaccination status, age at sexual debut, and smoking, and no clinical outcome data (cytology, histology, or cervical lesions) were available. Because HPV testing was performed both for routine screening and for clinical indications—which could not be reliably distinguished in the records—the overall positivity may be somewhat higher than in a pure screening population; the reported rate should therefore be interpreted as the prevalence among tested individuals rather than a population-based screening estimate. In addition, Türkiye did not have a national HPV vaccination program during the study period, so the cohort is expected to be largely unvaccinated, which should be considered when comparing these findings with vaccinated populations. Only samples yielding a valid, complete result for all three reporting channels were included; invalid or indeterminate tests were repeated as part of routine laboratory practice, but the laboratory information system did not retain a separate flag for these events, so the exact number of initially invalid and subsequently repeated samples could not be reconstructed.

In the 21–24 age group, which predominantly consists of students, health literacy levels may be insufficient. In a study conducted by Taşçı et al., low levels of HPV awareness were reported among university students [24]. A similar finding was reported by Borlu et al., who observed that only 36% of participants were aware of HPV screening and vaccination [25]. Considering that this study was conducted in 2016, it highlights the need for more recent and comprehensive awareness studies. As social awareness increases and vaccination programs become more widespread, a decline in HPV positivity rates can be anticipated. Additionally, it has been reported in the literature that HPV positivity tends to decrease with increasing age [26].

One of the limitations of our study is that the data were obtained from a single city. However, this city is one of the three most populous cities in the country, and the hospital where the study was conducted is among the highest-volume healthcare centers nationwide. Nevertheless, the age-specific HPV positivity distribution may differ from that of the general population. Furthermore, differences in the age of sexual debut, as well as sociocultural attitudes and behaviors across countries, can influence not only HPV positivity rates but also the age groups in which infections are detected. Therefore, multicenter studies with larger sample sizes are clearly needed. We also believe that more educational and awareness-focused studies on HPV should be conducted.

Interestingly, despite the smaller number of patients in the 21–24 age group in our study, this group demonstrated the highest HPV positivity rate. This suggests that preventive healthcare services and awareness initiatives may be particularly impactful in this population. Moreover, the widespread use of HPV vaccines is expected to reduce the number of cases across all age groups.

## 5. Conclusions

In conclusion, HPV remains the most significant risk factor for cervical cancer. Early detection of HPV infection through screening programs has been reported to contribute to reducing the incidence of HPV-related malignancies. Therefore, early identification of HPV positivity is of paramount importance, as it can also enhance the effectiveness of HPV vaccination programs. As this was a single-center, hospital-based study, our findings should be interpreted as regional rather than nationally representative; they nonetheless provide age-specific prevalence estimates from a large cohort. We believe that our study, with its large patient cohort, will serve as a valuable reference for future research in this field.

## Figures and Tables

**Table 1:** Age groups and patient numbers.

	Number (*n*)	Percentage (%)
Age groups		
21–24	2140	4.0
25–29	3975	7.5
30 and older	46,815	88.4

**Table 2:** Numbers and percentages of positive screenings by HPV genotype groups.

	Number (*n*)	Percentage (%)
HPV 16 only		
Positive	2118	4.0
HPV 18 only		
Positive	528	1.0
HR-HPV other		
Positive	5693	10.8
HPV 16 + HPV 18		
Co-positive	237	0.4
HPV 16 + HR-HPV other		
Co-positive	1197	2.3
HPV 18 + HR-HPV other		
Co-positive	513	1.0
HPV 16 + HPV 18 + HR-HPV other		
Triple-positive	145	0.3
Results		
Negative	42,499	80.3
Positive	10,431	19.7

**Table 3:** Differences in relevant parameters across age groups.

	21–24 Years (*n* = 2140)	25–29 Years (*n* = 3975)	30 Years and Older (*n* = 46,815)	*p* ***
	*n* (%)	*n* (%)	*n* (%)	
HPV 16 only				
Positive	110 (5.1)	217 (5.5)	1791 (3.8)	<0.001 **
HPV 18 only				
Positive	36 (1.7)	50 (1.3)	442 (0.9)	<0.001 **
HR-HPV other				
Positive	416 (19.4)	629 (15.8)	4648 (9.9)	<0.001 **
HPV 16 + HPV 18				
Co-positive	37 (1.7)	54 (1.4)	146 (0.3)	<0.001 **
HPV 16 + HR-HPV other				
Co-positive	188 (8.8)	208 (5.2)	801 (1.7)	<0.001 **
HPV 18 + HR-HPV other				
Co-positive	83 (3.9)	95 (2.4)	335 (0.7)	<0.001 **
HPV 16 + HPV 18 + HR-HPV other				
Triple-positive	26 (1.2)	35 (0.9)	84 (0.2)	<0.001 **
Total				
Negative	1244 (58.1)	2687 (67.6)	38,568 (82.4)	<0.001 **
Positive	896 (41.9)	1288 (32.4)	8247 (17.6)	

** *p* < 0.01; *** Chi-square test.

**Table 4:** All samples positive for HPV16, HPV18 in any combination.

	Number (*n*)	Percentage (%)
HPV 16		
Positive	3697	7.0
HPV 18		
Positive	1423	2.7

## Data Availability

The datasets generated and/or analyzed during the current study are available from the corresponding author on reasonable request.

## Supplementary Materials

The following supporting information can be downloaded at https://www.mdpi.com/article/10.3390/jcm15166323/s1, Table S1: Point prevalence with 95% confidence intervals (Wilson score method), computed from the reported counts (total *n* = 52,930).

## Abbreviations

The following abbreviations are used in this manuscript:

HPV	Human papilloma virus
HR	High Risk
