# Supplementary material for: The Prevalence of Human Papilloma Virus in Turkish Women
Source: J Clin Med. 2026 Aug 16;15(16):6323. doi: 10.3390/jcm15166323 (PMC13513448; doi:10.3390/jcm15166323)
Supplement: Supplementary file 1 [file jcm-15-06323-s001.zip › jcm-4468298-supplementary.pdf]

Supplementary Materials

The Prevalence of Human Papilloma Virus in Turkish Women
Manuscript ID: jcm-4468298

Table S1. Point prevalence with 95% confidence intervals (Wilson score method), computed from the reported counts (total N = 52,930).

Category / age group	n	Prevalence (%)	95% CI
Overall HR-HPV	10,431	19.7	19.4–20.0
HPV16 only	2,118	4.0	3.8–4.2
HPV18 only	528	1.0	0.9–1.1
Other HR only	5,693	10.8	10.5–11.0
HPV16 + HPV18	237	0.4	0.4–0.5
HPV16 + other HR	1,197	2.3	2.1–2.4
HPV18 + other HR	513	1.0	0.9–1.1
Triple (HPV16 + HPV18 + other HR)	145	0.3	0.2–0.3
**Overall HR-HPV positivity by age group**			
21–24 years	896/2,140	41.9	39.8–44.0
25–29 years	1,288/3,975	32.4	31.0–33.9
≥30 years	8,247/46,815	17.6	17.3–18.0

HR, high-risk; CI, confidence interval. Confidence intervals were calculated using the Wilson score method from the counts reported in the manuscript. The upper block reports the prevalence of each (mutually exclusive) genotype category in the full cohort; the lower block reports overall HR-HPV positivity within each age group.
